# The New York Code of Ethics

**Published:** 1882-08

**Authors:** 


### Editorial.

---

THE NEW YORK CODE OF ETHICS.

We learn that a large proportion of the county medical societies in the State of New York have already refused to sanction the revised code adopted by the State Society last February, thus rendering it quite probable that it will be repealed, or so modified as not to conflict with the national code, at the next annual meeting of the State Society.

It is certainly desirable that such a result should be reached; for one of the great benefits to be derived from a written code of ethics, depends upon its *nationality;* namely, its influence in unifying and harmonizing the profession by furnishing a common rule of action for all parties and sections. But the nationality of a code can neither be acquired nor preserved, unless its framing and revisions are both left in the hands of the American Medical Association, as the only national representative organization, the voting members of which are direct delegates from the State and local medical societies. If each State medical society should follow the example of that of New York, it is plain that all uniformity would be destroyed, and the conflicting ethical regulations would speedily render it impossible to preserve the harmony and representative character of a national organization. Consequently, the only proper course would be for any State society, the majority of whose members desire alterations of the ethical code, to instruct its delegates to the National Association to propose, in that body, such alterations as might be desired. That would give to the delegates from all the societies an opportunity to take part in the consideration and adoption or rejection of the changes proposed.

